# Effectiveness of Digital and Remote Health Interventions in Pediatric Populations From Underserved or Rural Areas: A Systematic Review of Randomized Controlled Trials

**DOI:** 10.1155/ijta/5586850

**Published:** 2026-02-16

**Authors:** Víctor Beltrán, Liliann Abarza, Pablo Acuña-Mardones, Iris Espinoza, Alain Chaple Gil, Constanza Morales-Gómez, Vanessa Campos-Bijit, Rafael Contador, Leonardo Díaz, Eduardo Fernández

**Affiliations:** ^1^ Clinical Investigation and Dental Innovation Center (CIDIC), Dental School and Center for Translational Medicine (CEMT-BIOREN), Universidad de La Frontera, Temuco, Chile, ufro.cl; ^2^ Department of Oral Rehabilitation, Dental School, Universidad de La Frontera, Temuco, Chile, ufro.cl; ^3^ Doctoral Program in Morphological Sciences, Universidad de La Frontera, Temuco, Chile, ufro.cl; ^4^ Department of Pathology and Oral Medicine, Faculty of Dentistry, University of Chile, Santiago, Chile, uchile.cl; ^5^ Facultad de Ciencias de la Salud, Universidad Autónoma de Chile, Santiago, Chile, uautonoma.cl; ^6^ Laboratory of Nanobiomaterials, Research Institute of Dental Sciences, Faculty of Dentistry, Universidad de Chile, Santiago, Chile, uchile.cl; ^7^ Núcleo Milenio Bioproductos, Genómica y Microbiología Ambiental (BioGEM), Valparaíso, Chile; ^8^ Department of Restorative Dentistry, Facultad de Odontologia, Universidad de Chile, Santiago, Chile, uchile.cl; ^9^ Department of Stomatology, Faculty of Dentistry, Universidad de Sevilla, Sevilla, Spain, us.es; ^10^ Perioplastic Institute, Santiago, Chile; ^11^ Prosthetics Department, Faculty of Dentistry, University of Chile, Santiago, Chile, uchile.cl; ^12^ Instituto de Ciencias Biomédicas, Universidad Autónoma de Chile, Santiago, Chile, uautonoma.cl

**Keywords:** mHealth, pediatric care, rural health services, telehealth, underserved populations

## Abstract

**Background:**

Children living in underserved and rural areas experience significant barriers to healthcare access due to geographic isolation, health workforce shortages, and systemic inequities. Digital and remote health interventions such as telehealth, telemental health (TMH), and mobile health (mHealth) offer promising strategies to improve pediatric health outcomes in these contexts. However, the extent of their effectiveness remains insufficiently examined through high‐quality evidence.

**Methods:**

A systematic review was conducted in accordance with PRISMA 2020 guidelines and structured using the PROPS framework. Five databases (PubMed, Scopus, Web of Science, Embase, and Cochrane Library) were searched for randomized controlled trials (RCTs) published until May 2025. Eligible studies targeted children (0–17 years) in underserved or rural settings and evaluated digital or remote interventions versus standard care. Data were extracted on study design, population, intervention modality, outcomes, and implementation characteristics. Risk of bias was assessed using the Cochrane RoB 2.0 tool.

**Results:**

Eleven RCTs were included, covering interventions for obesity, asthma, ADHD, diabetes, oral health, and neonatal care. Telehealth interventions improved behavioral and biometric outcomes (e.g., BMI *z*‐score, and adherence), particularly in the United States. TMH showed high fidelity and effectiveness for ADHD management. mHealth interventions in low‐ and middle‐income countries enhanced referral rates, service coverage, and caregiver engagement. Most studies were rated low risk of bias, though few incorporated economic or equity analyses.

**Conclusions:**

Digital health interventions are effective and feasible for improving pediatric outcomes in underserved settings. Future research should emphasize long‐term impact, cost‐effectiveness, and equitable access to ensure sustainable and inclusive digital healthcare delivery.

## 1. Introduction

Pediatric populations in underserved and rural areas face persistent and multifactorial barriers to accessing timely, high‐quality healthcare. These challenges include geographic remoteness, shortages of specialized providers, transportation difficulties, low digital literacy, and structural inequities rooted in social determinants of health [1, 2]. Children living in such contexts are disproportionately affected by preventable or manageable conditions such as asthma, obesity, developmental disorders, and infectious diseases which often remain underdiagnosed or poorly managed due to limited service availability [3–5], as well as a lack of specialists across various areas of healthcare. As a result, there is growing interest in leveraging digital and remote health technologies to bridge these access gaps [6, 7].

In this review, underserved populations are defined as communities experiencing structural barriers to healthcare access, including limited availability of clinicians, geographic isolation, socioeconomic disadvantage, and reduced digital infrastructure. Rural populations were defined according to each study′s operational criteria, typically referring to areas with low population density, substantial distance from tertiary health services, and designation as rural or remote by national or regional authorities. We considered underserved and rural populations jointly because they frequently overlap in structural disadvantages—such as restricted specialist access, limited broadband coverage, and fewer healthcare resources—regardless of whether they are located in high‐income countries (HICs) or low‐ and middle‐income countries (LMICs).

Digital health interventions including telehealth [8], telemental health (TMH) [9, 10], and mobile health (mHealth) [11, 12] offer the potential to transform pediatric healthcare delivery in resource‐constrained settings. These technologies enable remote consultation, behavior modification, disease management, and health education through modalities such as videoconferencing, text messaging, mobile apps, and decision‐support tools [13, 14]. Although these interventions are increasingly deployed worldwide, their effectiveness in pediatric populations, particularly those living in marginalized environments, requires systematic evaluation using robust study designs such as randomized controlled trials (RCTs).

Several systematic reviews have examined digital health in children [15–19], but few have focused specifically on underserved or rural populations [20, 21], which are often excluded or underrepresented in major trials. Moreover, existing reviews typically pool heterogeneous studies without disaggregating results by context, age, or modality, limiting their applicability to vulnerable settings. In addition, many prior reviews have not incorporated implementation dimensions (e.g., acceptability, fidelity, and equity), despite their critical importance in digital health scalability [22, 23]. In addition, it is essential to consider the future research directions and limitations of these studies in order to design telehealth strategies that align with the needs of this population and are relevant to diverse geographical and cultural settings worldwide. Although LMICs and HICs differ in economic capacity and health system organization, underserved and rural communities across these contexts share key structural vulnerabilities, including limited access to pediatric specialists, transportation barriers, reduced digital readiness, and lower health literacy. These shared constraints justify a combined analytical approach focused on contextual disadvantage rather than national income classification. This perspective aligns with global digital health frameworks emphasizing equity across heterogeneous but similarly marginalized settings.

Delivering digital health interventions in underserved and rural settings is further challenged by structural limitations in digital infrastructure. For example, broadband penetration in rural areas remains substantially lower than in urban regions, with global estimates indicating that rural households are 30%–50% less likely to have stable high‐speed internet access, depending on the country. Families living in poverty also report lower device ownership and greater instability in mobile data access, which contributes to the well‐documented “digital divide.” These constraints imply that the populations who may benefit most from telehealth are often those least able to access it, reinforcing the need to evaluate digital interventions specifically within disadvantaged pediatric contexts.

To address these gaps, we conducted a systematic review of RCTs [24] evaluating the effectiveness of digital and remote health interventions for children and adolescents in underserved or rural settings. We structured our review using the PROPS [25] (population, reporting of outcomes, procedures, and study design) framework to guide inclusion and analysis. In addition to summarizing clinical effectiveness, we synthesized information on implementation barriers, user engagement, and contextual adaptability.

Our objective was to answer the following research question: “Are digital and remote health interventions effective in improving clinical and behavioral outcomes among children living in underserved or rural areas, compared with usual care?”

By focusing exclusively on high‐quality trials and incorporating equity‐ and context‐sensitive analysis, this review is aimed at informing clinicians, researchers, and policymakers on the potential and limitations of digital interventions in pediatric care for vulnerable populations.

## 2. Materials and Methods

This systematic review was conducted in accordance with the PRISMA [24] (Preferred Reporting Items for Systematic Reviews and Meta‐Analyses) 2020 guidelines (Page et al. 2021), and structured using the PROPS [25] framework, which emphasizes key dimensions of systematic evidence synthesis in digital health: population, reporting of outcomes, procedures, and study design. The protocol was registered in PROSPERO (CRD420251066179), all methods were predefined, and the review process followed rigorous standards of transparency and reproducibility.

### 2.1. Eligibility Criteria

Studies were included if they met the following criteria (1): RCT design (2); pediatric population aged 0–18 years (3); participants living in underserved or rural areas, as explicitly defined by authors or inferred from context (e.g., low‐resource settings, health professional shortage areas, and geographically isolated regions) (4); evaluation of a digital or remote health intervention (telehealth, TMH, mHealth, or hybrid) (5); comparison against usual care, waitlist control, or standard face‐to‐face care; and (6) reporting of at least one clinical, behavioral, or service delivery outcome relevant to pediatric care. Studies were excluded if they involved simulation, protocols without results, or if the digital component was not a core part of the intervention. Eligible health conditions included both chronic diseases (e.g., obesity, asthma, diabetes, and ADHD) and acute or episodic conditions managed in community or primary care settings (e.g., neonatal complications and acute childhood illnesses). No restrictions were placed on condition type, provided the intervention included a digital or remote health component and targeted pediatric populations in underserved or rural settings.

The “user” of the digital intervention could be the child, caregiver, community health worker (CHW), teacher, or healthcare provider, depending on the intervention design. Interventions were eligible if the primary target of the health outcome was the child, even if the digital tool was used primarily by caregivers or health workers (e.g., decision‐support systems and parent‐mediated telehealth coaching).

A study was classified as conducted in a rural or underserved area if this designation was explicitly reported by the authors, or if contextual descriptors indicated limited resources (e.g., health professional shortage areas, remote communities, low‐income rural regions, or districts with documented service gaps). Interventions delivered simultaneously in mixed urban–rural contexts were included only if outcome data specifically pertained to the underserved or rural subpopulation.

Primary outcomes of interest included clinical indicators (e.g., BMI *z*‐scores, glycemic control, asthma technique, and neonatal referral timeliness) and validated behavioral or psychological outcomes when applicable. Secondary outcomes included service delivery metrics such as adherence, session completion, referral accuracy, caregiver engagement, and acceptability. These outcome domains were selected a priori to reflect both clinical effectiveness and implementation performance of digital health interventions in underserved pediatric populations.

Comparators labelled as “usual care” varied across studies and could include standard in‐person clinical visits, group education sessions, routine CHW practices, or no structured follow‐up beyond standard health system provisions. To maintain transparency, the specific components of “usual care” for each RCT were extracted and reported. Given this heterogeneity, interpretations of comparative effectiveness were made cautiously.

### 2.2. Search Strategy

A comprehensive electronic search was conducted in five major databases: PubMed, Scopus, Web of Science, Embase, and the Cochrane Central Register of Controlled Trials (CENTRAL). The search covered the period until May 2025 and used a combination of Medical Subject Headings (MeSH) and free‐text terms related to pediatrics, telemedicine, digital health, remote care, underserved populations, and randomized trials. No language restrictions were applied. The full search strategy is available (File S1). Reference lists of included articles and relevant reviews were also manually screened for additional studies.

### 2.3. Study Selection

All search results were imported into Rayyan QCRI [26], a web‐based systematic review tool. Two reviewers independently screened titles and abstracts for relevance, followed by full‐text reviews of potentially eligible studies. Discrepancies were resolved through consensus with a third reviewer. Interrater agreement at the full‐text screening stage was high (Cohen^′^s kappa = 0.84).

### 2.4. Data Extraction and Synthesis

Intervention modalities were categorized into three domains: telehealth, TMH, and mHealth—based on established digital health taxonomies. Telehealth referred to synchronous video‐based clinical or behavioral care; TMH included remote psychiatric or psychological services; and mHealth comprised mobile or SMS‐based tools, including decision‐support applications for CHWs. These categories enabled consistent comparison across highly heterogeneous interventions and aligned with current WHO and NIH digital health classification frameworks.

Data were extracted using a standardized form covering study setting, country, sample size, participant characteristics, type of digital intervention, modality (e.g., video, SMS, and app), comparison group, primary outcomes, follow‐up duration, and implementation metrics (e.g., adherence and acceptability). Extracted data were cross‐checked by a second reviewer to ensure accuracy.

Due to substantial heterogeneity in intervention types, outcomes, and follow‐up durations, a quantitative meta‐analysis was not feasible. Instead, a narrative synthesis approach was applied, categorizing results by intervention modality (telehealth, TMH, and mHealth), outcome domain (clinical, behavioral, and service‐related), and geographical context (high‐income vs. LMICs). Special attention was paid to reported implementation barriers and equity considerations.

### 2.5. Risk of Bias Assessment

Risk of bias in included studies was independently assessed by two reviewers using the Cochrane RoB 2.0 tool [27]. This tool evaluates five domains: randomization process, deviations from intended interventions, missing outcome data, outcome measurement, and selection of reported results. Each domain was rated as “low risk,” “some concerns,” or “high risk.” Overall study quality was classified accordingly.

### 2.6. Reporting Standards and Ethical Considerations

This review adhered to the PRISMA 2020 checklist for transparent reporting. Since no human subjects were directly involved, institutional review board (IRB) approval was not required. The PROPS framework guided the analytical structure of this review. Population informed inclusion criteria emphasizing structural disadvantage. Reporting of outcomes ensured extraction of all clinical, behavioral, and service delivery indicators. Procedures shaped the classification of digital modalities, implementation factors, and contextual conditions. Study design ensured inclusion of only RCTs and guided the risk‐of‐bias assessment. This structured approach enhanced consistency in synthesis across heterogeneous interventions.

## 3. Results

### 3.1. Study Selection and General Characteristics

**Figure 1:**
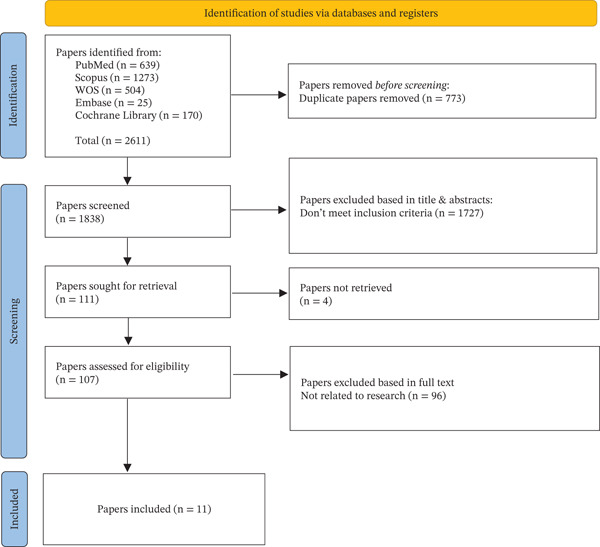
Identification of studies via databases and registers (PRISMA flow diagram).

From a total of 1542 records initially identified, 11 RCTs [28–38] met the inclusion criteria after screening and full‐text review (Figure 1 and Table 1). These studies were published between 2001 and 2025 and represented both HICs such as the United States and Mexico (*n* = 7) and LMICs, including Guatemala, India, Zambia, and Kenya (*n* = 4). Sample sizes ranged from 60 to 400 participants. Most trials involved school‐aged children or adolescents, with interventions targeting chronic disease management, behavioral health, or early childhood care.

**Table 1:** Characteristics of the included randomized controlled trials.

Study	Country	Population	Intervention type	Comparator	Main outcomes
Bynum et al. [29]	United States	Adolescents with asthma in rural areas	Telepharmacy counseling via interactive compressed video	Written instructions	Improved metered‐dose inhaler (MDI) technique and higher patient satisfaction
Davis et al. [30]	United States	Children with obesity in rural areas	Family‐based telehealth behavioral intervention	Usual care	Reduced BMI *z*‐score and improved nutrition/physical activity
Davis et al. [31]	United States	Children with obesiy and their families in rural areas	Weekly multidisciplinary family‐based behavioral group via telemedicine	Telephone intervention	Sustained BMI improvement
Davis et al. [32]	United States	Children and parents with obesity in rural communities	Parent + child tele − sessions	Newsletter + family − based	High adherence; improved behavioral outcomes
Gallagher et al. [33]	United States	Obese children in rural schools	Basic pilot telehealth intervention	Usual care	Write the initial design and characteristicsof a randomized controlled trial designed to treat pediatric obesity in rural children.
Myers et al. [38]	United States	Children (223) with deficit/hyperactivity disorder (ADHD) in underserved areas	Telemental health (TMH) video teleconferencing (six sessions via video)	One‐session telepsychiatry and usual care	High protocol fidelity; > 95% completion rate.
Jewell et al. [34]	United States	Parents in rural areas with children diagnosed with Type 1 diabetes (T1D)	Parent + child telesessions	Standard care	Level of verbal participation by the father increased significantly over the 12 weeks. No changes in HbA1c.
Lopez Garcia et al. [35]	Mexico	Children in rural Kenya.	Remote oral hygiene education (mHealth) delivery via smartphones and session group.	In‐person health education	Testing an effective system using smartphones and group sessions to collect oral health evidence in rural populations in Kenya.
Martinez et al. [36]	Guatemala	Newborns and pregnant women in rural indigenous communities	mHealth decision‐support for TBAs.	Usual care by TBAs without mHealth system	Increased referrals to health facilities
Modi et al. [37]	India	Mothers and newborns in rural tribal communities	Community Health Worker (CHW) support via ImTeCHO mHealth platform.	Routine CHW care	Increased maternal, neonatal, and child Health (MNCH) service coverage, more postnatal visits, and improved breastfeeding practices
Biemba et al. [28]	Zambia	Children under five in rural households.	eCCM+ mobile decision support	Paper‐based IMCI tools.	Improved supportive supervision and supply chain management; increased appropriate treatment and referrals

Table 1 summarizes the general characteristics of the included studies, including geographic setting, target population, and intervention focus.

### 3.2. Intervention Modalities and Digital Tools

The included RCTs employed three main categories of digital interventions: telehealth, TMH, and mHealth.

• Telehealth interventions (*n* = 5) used synchronous video platforms to deliver pediatric obesity counseling (Davis et al. [30–32]), asthma education (Bynum et al. [29]), or diabetes management (Jewell et al. [34]). These studies were conducted in rural areas of the United States and Mexico and typically involved family‐based coaching models or direct clinical monitoring via video conferencing.
• TMH was evaluated in one large‐scale study focused on ADHD management (Myers et al. [38]), which combined centralized psychiatric support with caregiver‐guided behavioral therapy through remote sessions.
• mHealth tools (*n* = 5) were primarily deployed in LMICs (Guatemala, India, Zambia, and Kenya) and involved smartphone‐based decision‐support tools, SMS prompts, and CHW apps for maternal and child health (Martinez et al. [36]; Modi et al. [37]; Biemba et al. [28]; Lopez Garcia et al. [35]).

Table 2 provides a detailed breakdown of intervention modalities, digital platforms employed, if applicable, frequency of delivery, and technological requirements. The table highlights that although high‐income settings favored video‐based teleconsultation, LMICs relied more on low‐bandwidth, asynchronous tools compatible with limited infrastructure.

**Table 2:** summary of clinical outcomes by type of digital or remote intervention (revised).

Intervention type and focus	Study	Main findings	Effectiveness indicator
Telehealth–asthma management	Bynum et al. [29]	Significant improvement in MDI technique (*p* < 0.001); high acceptability; patient satisfaction similar to control (*p* = 0.132).	✓ Clear clinical improvement
Telehealth–pediatric obesity treatment	Davis et al. [30]	BMI *z*‐score unchanged between groups; both groups improved diet and activity; feasible and acceptable.	~ Mixed clinical effect; ✓ behavioral improvement
Telehealth–extended obesity care	Davis et al. [31]	High retention and satisfaction; no significant differences in BMIz or behavioral outcomes.	✗ No significant clinical effect; ✓ feasibility
Telehealth protocol–driven obesity management	Davis et al. [32]	Improvements in BMIz, physical activity, and diet; nurse involvement increased adherence.	✓ Clinical and behavioral improvement
Telehealth–baseline obesity data	Gallagher et al. [33]	Identified high‐risk population; demonstrated feasibility of remote weight‐management protocols.	• Feasibility emphasis
Telemental health–ADHD care coordination	Myers et al. [38]	High protocol fidelity; >95% session completion; significant reduction in ADHD symptoms via validated measures.	✓ Strong behavioral/clinical improvement
mHealth–Diabetes Self‐Management (T1D)	Jewell et al. [34]	No changes in HbA1c or TIR (baseline already optimal); improved parental autonomy, coaching behavior, and functional family goals.	~ No clinical change; ✓ behavioral/family improvement
mHealth–Oral Hygiene Education	Lopez Garcia et al. [35]	Feasible smartphone‐based oral health education; required linguistic and cultural adaptation.	• Feasibility; ~ early behavioral signals
mHealth–neonatal and maternal referrals	Martinez et al. [36]	Increased referrals; improved maternal complication detection; high feasibility in low‐resource settings.	✓ Service‐delivery improvement
mHealth–CHW monitoring for MNCH	Modi et al. [37]	Improved immunization, breastfeeding, and home‐visit coverage; strengthened CHW performance.	✓ Strong service‐delivery improvements
mHealth–under‐five pediatric care (ICCM)	Biemba et al. [28]	Faster caregiver care‐seeking; CHWs ~3× more likely to make appropriate referral decisions (*p* = 0.008).	✓ Clear service delivery and quality‐of‐care improvement

### 3.3. Clinical and Behavioral Outcomes

Most included trials reported positive clinical and behavioral outcomes in at least one domain.

• In the Davis et al. trials [30–32], children receiving telehealth‐based pediatric obesity treatment showed modest but significant reductions in BMI *z*‐scores, with effect sizes ranging from −0.09 to −0.21, depending on intervention duration and modality. For instance, Davis et al. [30] found no significant difference between telemedicine and in‐person interventions (*p* = 0.881), yet both groups improved significantly in dietary patterns (increased fruit/vegetable intake, reduced “red” food consumption). Session attendance exceeded 85% in all trials, indicating high feasibility and adherence.
• In the Bynum et al. [29] study on asthma management, adolescents receiving telepharmacy counseling via compressed video showed significant improvement in metered‐dose inhaler (MDI) technique compared with the control group (*p* < 0.001). However, no statistically significant differences in patient satisfaction were observed between groups (*p* = 0.132), with both reporting high satisfaction levels.
• Jewell et al. [34] evaluated a 12‐week telehealth occupational coaching program for rural families managing Type 1 diabetes. Although glycemic control (HbA1c) remained stable (no significant change), intervention families demonstrated greater achievement in family‐centered participation goals (*p* = 0.006), increased caregiver coaching talk (*p* = 0.034), and significantly improved EICRS scores (*p* < 0.001), reflecting enhanced parental efficacy in diabetes management.
• In the Myers et al. [38] CATTS trial, children with ADHD who received TMH intervention completed an average of 5.3 out of 6 planned sessions, and 96% of control participants completed at least one teleconsultation. The intervention group achieved > 95% adherence, and clinicians maintained high protocol fidelity, resulting in significant reductions in ADHD symptom severity as reported on validated outcome measures.
• mHealth interventions improved referral rates, treatment accuracy, and care‐seeking behavior. In Zambia, CHWs using the eCCM+ tool were nearly three times more likely to make correct treatment decisions compared with paper‐based protocols (aOR = 2.9; *p* = 0.008), and caregivers sought care faster (median: 1.3 vs. 2.2 days) (Biemba et al. [28]). In Guatemala, TBAs using smartphone decision support tools achieved a 28% higher referral rate and a 42% increase in timely neonatal care‐seeking compared with those using printed guidelines (*p* < 0.001) (Martínez et al. [36]).

Table 3 summarizes the clinical, behavioral, and service delivery outcomes reported across trials, categorized by intervention modality.

**Table 3:** Clinical recommendations from included randomized controlled trials.

Domain	Clinical recommendations
Telehealth for chronic conditions	Can be integrated into primary care to manage obesity, ADHD, and asthma in rural settings; requires caregiver engagement and structured protocols
Telemental health (TMH)	Effective for delivering behavioral and pharmacological interventions for pediatric ADHD; feasible with high fidelity via videoconferencing
mHealth for community health workers	Supports early detection and referral in neonatal and under‐5 care; beneficial when CHWs receive adequate digital training
mHealth for self‐management	Empowers adolescents with chronic conditions (e.g., diabetes) to improve self‐care and treatment adherence via mobile apps
Remote oral health education	Can improve hygiene practices in school‐aged children when in‐person instruction is not feasible; effective in LMIC contexts

### 3.4. Implementation Factors and Acceptability

Implementation data were variably reported but generally favorable. All studies reported moderate to high adherence rates, with several noting > 80% session completion. Acceptability was high among caregivers and children, particularly in interventions that were culturally adapted or supported by training. Barriers to implementation included internet connectivity issues, low digital literacy among CHWs or caregivers, and limitations in device access. In the Lopez Garcia et al. [35] trial, the use of smartphone‐based oral hygiene instruction in Kenya showed high feasibility but required local adaptation to language and literacy levels. Interestingly, none of the studies employed formal implementation science frameworks (e.g., RE‐AIM and CFIR), and none conducted economic evaluations or cost‐effectiveness analyses.

Table 4 presents implementation‐related findings, including adherence, technical barriers, cultural tailoring, and training components.

**Table 4:** Limitations and future research recommendations across digital health modalities.

Focus area	Key limitations identified in current evidence	Recommendations for future research
Telehealth (general)	Short‐term follow‐up in most RCTs; limited cost‐effectiveness data; heterogeneous intervention intensity; insufficient reporting of comparator (“usual care”).	✓ Conduct long‐term RCTs assessing sustained clinical effects.
		✓ Include cost‐effectiveness and implementation‐cost analyses.
		✓ Standardize comparator groups and outcome definitions.
Telemental health (TMH)	Evidence concentrated almost exclusively on ADHD; absence of studies on anxiety, depression, trauma, or behavioral dysregulation in underserved youth; limited culturally adapted TMH models.	✓ Expand TMH trials to broader pediatric mental health conditions.
		✓ Develop culturally adapted TMH protocols for underserved settings.
		✓ Evaluate differential effects by age, severity, and caregiver involvement.
mHealth (Community‐Level Tools)	Uncertain scalability; digital literacy gaps among CHWs and caregivers; variable infrastructure (connectivity, electricity, device availability); lack of formal implementation frameworks (RE‐AIM, CFIR).	✓ Test scalability in multi‐site or regional implementations.
		✓ Integrate digital literacy and training components into interventions.
		✓ Apply implementation science frameworks to evaluate fidelity, reach, and sustainability.
Self‐management and caregiver apps	Limited data on engagement decay over time; mixed evidence on direct clinical impact; few trials integrate apps with routine pediatric visits or EMR systems.	✓ Evaluate long‐term engagement and predictors of app adherence.
		✓ Combine self‐management apps with structured follow‐up or coaching.
		✓ Assess integration with electronic medical records and routine care pathways.
Digital equity (cross‐cutting)	Minimal reporting of socioeconomic, geographic, or digital‐access disparities; lack of equity‐derived outcome stratification; interventions may inadvertently widen the digital divide.	✓ Routinely collect and report equity indicators (SES, connectivity, device access, caregiver literacy).
		✓ Evaluate differential effectiveness in marginalized subgroups.
		✓ Design interventions that are bandwidth‐flexible and device‐agnostic.
Outcome measurement and standardization	Frequent use of nonvalidated or locally developed tools; poor comparability across studies; inconsistent behavioral, psychosocial, and service‐delivery metrics.	✓ Adopt validated pediatric outcome measures and core outcome sets.
		✓ Standardize digital health engagement metrics (fidelity, reach, and adherence).
		✓ Develop consensus measurement frameworks for global pediatric digital health.

### 3.5. Risk of Bias

**Figure 2:**
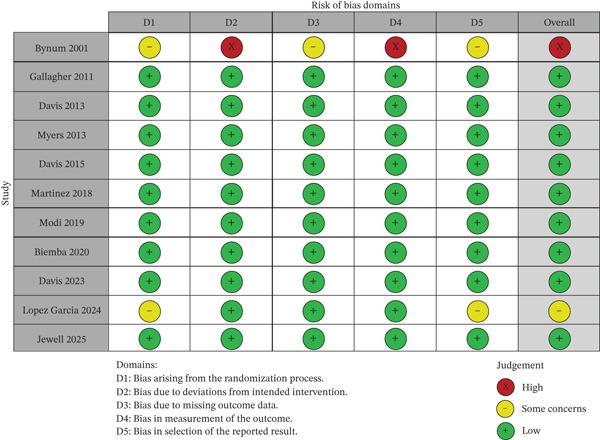
Risk of bias domains.

Risk of bias was rated as low in 8 of the 11 included studies, according to the Cochrane RoB 2.0 tool (Figure 2). Two studies [29, 35] were rated as having “some concerns”, mainly due to issues with randomization procedures, missing outcome data, or incomplete blinding of outcome assessment. One study [29] was assessed as having a high overall risk of bias, particularly in the domains of deviations from intended interventions and outcome measurement. Most trials adequately reported their primary outcomes and used robust randomization methods, supporting overall confidence in the evidence base.

## 4. Discussion

This systematic review of 11 RCTs provides evidence that digital and remote health interventions including telehealth, TMH, and mHealth are effective and feasible strategies to improve pediatric health outcomes in underserved and rural settings. Across high‐ and low‐income contexts, interventions led to improvements in clinical markers (e.g., BMI *z*‐scores and glycemic control), caregiver engagement, treatment adherence, and service utilization.

### 4.1. Interpretation of Key Findings

Telehealth interventions demonstrated robust effects in managing chronic pediatric conditions such as obesity, asthma, and diabetes in high‐income settings. The trials by Davis et al. [32] showed consistent improvements in weight‐related outcomes when telehealth was combined with caregiver involvement and behavior‐focused coaching. These findings reinforce the potential of synchronous video models to support longitudinal disease management in rural populations with limited access to pediatric specialists. Moreover, evidence suggests that telemonitoring of pediatric patients with obesity may be an effective strategy to enhance behavioral guidance and facilitate access to specialist care through both synchronous and asynchronous modalities.

TMH, evaluated in the large‐scale study by Myers et al. [38], proved highly effective in extending psychiatric care to children with ADHD. Notably, this model achieved high fidelity and completion rates despite operating in underserved regions. These results support TMH as a scalable option for behavioral health, which remains a critical gap in rural pediatric services.

In contrast, mHealth strategies were more prominent in LMICs and served as low‐cost, offline‐compatible solutions for frontline health workers. Studies from Zambia, India, and Guatemala [28, 36, 37] revealed significant gains in referral accuracy, postnatal visit coverage, and caregiver responsiveness. The ability of mHealth to function in resource‐constrained environments with minimal infrastructure demonstrates its adaptability and relevance for global pediatric health delivery.

Despite the overall positive outcomes, the included interventions varied markedly in duration (ranging from 2 weeks to 12 months), intensity (from single‐session consults to weekly structured sessions), and technological sophistication from SMS‐based messaging to smartphone‐enabled clinical decision support systems and HIPAA‐compliant video platforms. This heterogeneity, although reflective of necessary context‐specific adaptations across settings and populations [28, 36], limits the comparability of results and weakens external generalizability. For example, some trials used simple text messaging tools for follow‐up, whereas others relied on multilayered teleconsultation platforms integrated with electronic health records [39].

Furthermore, outcome measurement strategies were inconsistently applied, particularly in evaluating behavioral change, adherence, and service delivery improvements. Many studies used nonvalidated or custom‐built indicators without standardized thresholds, complicating pooled interpretation and replication. As emphasized by Jin et al. [39], this underscores the urgent need to develop and adopt unified digital health evaluation frameworks that ensure methodological consistency while retaining flexibility for diverse clinical and geographic contexts.

Moreover, standardization in outcome measurement remains limited, particularly for behavioral, psychosocial, and service delivery domains. Studies often relied on custom‐developed or proxy metrics—such as caregiver‐reported adherence or user satisfaction—rather than validated tools, hindering replicability [39, 40]. For example, Taher et al. [41] noted the absence of unified metrics for evaluating engagement in digital ADHD therapies, complicating comparative effectiveness research. As these reviews highlight, a harmonized framework for evaluating pediatric digital health interventions is urgently needed, particularly to integrate clinical outcomes, user experience, and implementation fidelity across diverse populations and platforms. The recurrent use of nonvalidated or locally developed outcome measures across studies underscores a foundational measurement gap that precedes and justifies the call for unified digital health evaluation frameworks. Standardized pediatric outcome sets would allow consistent reporting, facilitate comparison across settings, and support the development of implementation–effectiveness hybrid models.

Importantly, several trials reported improvements in both intervention and usual‐care groups, reflecting the possibility that structured follow‐up alone regardless of digital modality may benefit families living in underserved environments. Understanding the exact nature of usual care is therefore essential for interpreting intervention effects. Future RCTs should standardize reporting of comparator conditions to improve interpretability and enable more precise comparative effectiveness analyses.

### 4.2. Implementation and Scalability Challenges

Although all studies reported high feasibility and acceptability, several implementation barriers were recurrent. Digital infrastructure challenges such as poor internet connectivity, device unavailability, and inconsistent electricity were cited across LMICs and some rural areas in HICs [42–44]. Low digital literacy among caregivers or CHWs also emerged as a barrier, especially in settings where technological training was limited [45, 46].

Another major limitation was the absence of economic analyses. None of the included RCTs assessed the cost‐effectiveness or budgetary impact of digital interventions compared with standard care. This omission limits policy translation, especially in publicly funded health systems and LMICs where investment decisions must be evidence‐based. Without robust economic data, the scalability of otherwise effective interventions remains uncertain.

None of the included RCTs explicitly reported using codesign or participatory methods involving children, caregivers, or community stakeholders during intervention development. This represents an important limitation, given that codesign improves cultural fit, usability, and digital engagement. Future pediatric digital health trials should integrate structured codesign approaches as part of their development and implementation processes.

### 4.3. Equity and Inclusion Gaps

Although all studies targeted underserved settings, few explicitly analyzed differential effects by socioeconomic status, gender, ethnicity, or baseline digital access. This represents a significantly missed opportunity. Without equity‐disaggregated data, it is unclear whether interventions reach the most marginalized subgroups or inadvertently widen the digital divide. For example, children from households without smartphones or stable internet may be systematically excluded from both synchronous and asynchronous interventions.

To ensure equitable digital transformation in pediatric care, future studies should incorporate equity‐focused implementation metrics and recruitment strategies that prioritize marginalized populations. Additionally, codesign with local stakeholders, including caregivers, adolescents, and CHWs, can enhance cultural fit and intervention acceptability.

Equity considerations were insufficiently addressed in most trials. From a PROPS “population” perspective, essential indicators—such as baseline digital access, socioeconomic status, disability status, gender, and ethnicity—were rarely stratified in analyses. Future trials should routinely collect and report equity‐disaggregated outcomes to evaluate whether digital interventions mitigate or exacerbate disparities.

Important gaps emerged in the distribution of conditions and age groups represented. Most interventions targeted school‐aged children with chronic conditions, whereas infants, toddlers, and adolescents were comparatively underrepresented. Likewise, no RCTs addressed digital interventions for developmental disabilities, infectious diseases beyond community case management, or oral health beyond a single study. These gaps highlight priority areas for future research.

### 4.4. Limitations of the Review

This review has limitations. First, heterogeneity in interventions and outcome measures precluded meta‐analysis. Second, despite extensive database coverage, publication bias remains possible, particularly as successful digital trials may be more likely to be published. Lastly, most included studies were conducted in specific national contexts (e.g., United States and India), which may limit generalizability to other regions.

Given that only 11 randomized trials met inclusion criteria and that these studies spanned diverse clinical conditions, age groups, and health system contexts, the generalizability of conclusions is necessarily limited. The heterogeneity of intervention modalities and outcome measures further constrains the extent to which overarching conclusions can be drawn. Therefore, our findings should be interpreted as evidence of potential effectiveness rather than definitive confirmation across all pediatric conditions or settings.

### 4.5. Implications for Policy and Research

For digital health to fulfill its promise in underserved pediatric populations, future RCTs must integrate long‐term follow‐up, economic evaluations, and equity monitoring. Policymakers should invest in infrastructure, digital training, and health system integration, whereas funding agencies should prioritize studies that apply implementation science and participatory design. Future research should adopt standardized pediatric digital health metrics, including validated behavioral rating scales (e.g., SDQ and Vanderbilt scales), functional measures, core outcome sets for chronic pediatric conditions, and uniform implementation indicators such as fidelity, acceptability, reach, and equity‐sensitive measures. Standardization would substantially improve comparability and policy translation. Future research should consider comparative designs that explicitly contrast rural versus nonrural or underserved versus adequately‐served populations. Such comparisons would enhance understanding of whether digital interventions yield disproportionate benefits in structurally disadvantaged settings and would expand the pool of eligible studies by not restricting samples exclusively to rural or underserved groups.

Although the evidence reviewed suggests meaningful promise for digital interventions in pediatric underserved settings, the small number of available RCTs, variations in intervention intensity, and lack of standardized measurement tools preclude definitive conclusions. The current evidence base should be viewed as preliminary but encouraging, underscoring the need for more rigorous, larger‐scale, and context‐sensitive trials.

## 5. Conclusions

This systematic review demonstrates that digital and remote health interventions, including telehealth, TMH, and mHealth, are effective and feasible strategies for improving pediatric health outcomes in underserved and rural populations. The reviewed RCTs showed consistent benefits across clinical, behavioral, and service delivery domains. However, challenges related to infrastructure, digital literacy, and lack of economic and equity evaluations persist. To maximize long‐term impact, future research should integrate cost‐effectiveness analyses, implementation of science frameworks, and equity‐focused designs. Health systems must support digital integration through policy alignment, investment in digital infrastructure, and culturally responsive care models. Scalable and inclusive digital health interventions can play a critical role in advancing pediatric health equity globally. In this context, there is a pressing need for further research to evaluate the impact of telehealth strategies, with a particular focus on pediatric populations that face access barriers to general and specialized healthcare services across diverse geographical and cultural settings.

## Funding

No funding was received for this manuscript.

## Ethics Statement

The authors have nothing to report.

## Consent

The authors have nothing to report.

## Conflicts of Interest

The authors declare no conflicts of interest.

## Supporting Information

Additional supporting information can be found online in the Supporting Information section.

## Supporting information


**Supporting Information 1** File S1: Complete search strategies for all databases (PubMed, Scopus, Web of Science, Embase, and Cochrane Library), including MeSH terms, title/abstract keywords, and filters applied.

**Supporting Information 2** File S2: Figure legends for Figure 1 (PRISMA flow diagram) and Figure 2 (Risk of Bias Summary), describing the selection process and quality assessment of included randomized controlled trials.

## Data Availability

The data that support the findings of this study are available from the corresponding author upon reasonable request.
