# Supplementary material for: Effectiveness of Digital and Remote Health Interventions in Pediatric Populations From Underserved or Rural Areas: A Systematic Review of Randomized Controlled Trials
Source: Int J Telemed Appl. 2026 Feb 16;2026:5586850. doi: 10.1155/ijta/5586850 (PMC12908196; doi:10.1155/ijta/5586850)
Supplement: Supplementary file 1 — Supporting Information 1 File S1: Complete search strategies for all databases (PubMed, Scopus, Web of Science, Embase, and Cochrane Library), including MeSH terms, title/abstract keywords, and filters applied. [file IJTA-2026-5586850-s002.docx]

| **Database** | **Formulation** | **Filters** |
| --- | --- | --- |
| Pubmed | (  "Rural Population"[MeSH Terms] OR  "Medically Underserved Area"[MeSH Terms] OR  "Health Services Accessibility"[MeSH Terms] OR  "Rural Health"[MeSH Terms] OR  "rural areas"[Title/Abstract] OR  "remote areas"[Title/Abstract] OR  "underserved populations"[Title/Abstract] OR  "geographically isolated"[Title/Abstract] OR  "hard-to-reach areas"[Title/Abstract] OR  "rural communities"[Title/Abstract]  )  AND  (  "Telemedicine"[MeSH Terms] OR  "Teledentistry"[MeSH Terms] OR  "Remote Consultation"[MeSH Terms] OR  "Mobile Health Units"[MeSH Terms] OR  "Electronic Health Records"[MeSH Terms] OR  "Telecommunications"[MeSH Terms] OR  "telehealth"[Title/Abstract] OR  "telemedicine"[Title/Abstract] OR  "teledentistry"[Title/Abstract] OR  "e-health"[Title/Abstract] OR  "mobile health"[Title/Abstract] OR  "teleconsultation"[Title/Abstract] OR  "remote monitoring"[Title/Abstract] OR  "digital health"[Title/Abstract]  )  AND  (  "Treatment Outcome"[MeSH Terms] OR  "Program Evaluation"[MeSH Terms] OR  "Outcome Assessment, Health Care"[MeSH Terms] OR  "Quality of Health Care"[MeSH Terms] OR  "Effectiveness"[Title/Abstract] OR  "treatment outcome"[Title/Abstract] OR  "intervention effectiveness"[Title/Abstract] OR  "clinical effectiveness"[Title/Abstract] OR  "access improvement"[Title/Abstract] OR  "coverage improvement"[Title/Abstract] OR  "service performance"[Title/Abstract] OR  "quality of care"[Title/Abstract] OR  "health service utilization"[Title/Abstract]  ) | Filters applied: Clinical Study, Clinical Trial, Controlled Clinical Trial, Randomized Controlled Trial. |
| Scopus | (TITLE-ABS-KEY("rural area*" OR "remote area*" OR "rural population*" OR "rural communit*" OR "hard-to-reach area*" OR "underserved population*" OR "geographically isolated"))  AND  (TITLE-ABS-KEY("telehealth" OR "telemedicine" OR "teledentistry" OR "e-health" OR "mobile health" OR "teleconsultation" OR "remote monitoring" OR "digital health"))  AND  (TITLE-ABS-KEY("effectiveness" OR "treatment outcome*" OR "intervention effectiveness" OR "clinical effectiveness" OR "program evaluation" OR "healthcare access" OR "access improvement" OR "coverage improvement" OR "health service utilization" OR "service performance" OR "quality of care")) | AND ( LIMIT-TO ( DOCTYPE , "ar" ) ) |
| WoS | TS=("rural area*" OR "remote area*" OR "rural population*" OR "rural communit*" OR "hard-to-reach area*" OR "underserved population*" OR "geographically isolated")  AND  TS=("telehealth" OR "telemedicine" OR "teledentistry" OR "e-health" OR "mobile health" OR "teleconsultation" OR "remote monitoring" OR "digital health")  AND  TS=("effectiveness" OR "treatment outcome*" OR "intervention effectiveness" OR "clinical effectiveness" OR "program evaluation" OR "healthcare access" OR "access improvement" OR "coverage improvement" OR "health service utilization" OR "service performance" OR "quality of care") | Refined By:Document Types: Article |
| Embase | ('rural area*' OR 'remote area*' OR 'rural population*' OR 'rural communit*' OR 'hard-to-reach area*' OR 'underserved population*' OR 'geographically isolated')  AND  ('telehealth' OR 'telemedicine' OR 'teledentistry' OR 'e-health' OR 'mobile health' OR 'teleconsultation' OR 'remote monitoring' OR 'digital health')  AND  ('effectiveness' OR 'treatment outcome*' OR 'intervention effectiveness' OR 'clinical effectiveness' OR 'program evaluation' OR 'healthcare access' OR 'access improvement' OR 'coverage improvement' OR 'health service utilization' OR 'service performance' OR 'quality of care') | AND 'randomized controlled trial'/de |
| Cochrane Library  170 | ("rural area*" OR "remote area*" OR "rural population*" OR "hard-to-reach area*" OR "underserved population*" OR "geographically isolated")  AND  ("telehealth" OR "telemedicine" OR "teledentistry" OR "e-health" OR "mobile health" OR "teleconsultation" OR "remote monitoring" OR "digital health")  AND  ("effectiveness" OR "treatment outcome*" OR "program evaluation" OR "access improvement" OR "coverage improvement" OR "health service utilization" OR "quality of care") |  |
